# Stratification-Based Outlier Detection over the Deep Web

**DOI:** 10.1155/2016/7386517

**Published:** 2016-05-25

**Authors:** Xuefeng Xian, Pengpeng Zhao, Victor S. Sheng, Ligang Fang, Caidong Gu, Yuanfeng Yang, Zhiming Cui

**Affiliations:** ^1^Department of Computer Science and Technology, Soochow University, Suzhou, Jiangsu 215002, China; ^2^School of Computer Engineering, Suzhou Vocational University, Suzhou, Jiangsu 215104, China; ^3^Computer Science Department, University of Central Arkansas, Conway, AR 72035, USA

## Abstract

For many applications, finding rare instances or outliers can be more interesting than finding common patterns. Existing work in outlier detection never considers the context of deep web. In this paper, we argue that, for many scenarios, it is more meaningful to detect outliers over deep web. In the context of deep web, users must submit queries through a query interface to retrieve corresponding data. Therefore, traditional data mining methods cannot be directly applied. The primary contribution of this paper is to develop a new data mining method for outlier detection over deep web. In our approach, the query space of a deep web data source is stratified based on a pilot sample. Neighborhood sampling and uncertainty sampling are developed in this paper with the goal of improving recall and precision based on stratification. Finally, a careful performance evaluation of our algorithm confirms that our approach can effectively detect outliers in deep web.

## 1. Introduction

As a result of the rapid development of e-commerce, the deep web has been increasingly valued by data mining researchers in recent years. The deep web, which is termed to make a contrast with the surface web, refers to data sources with back-end databases that are only accessible through a query interface [1]. Currently, the vast majority of research on the deep web considers the problem on how to build an interactive query system or a vertical search system using data integration technologies [2, 3]. Seldom have articles conducted data mining over the deep web. Mining deep web data sources has unique challenges. The fundamental reason is that the acquisition of deep web data sources is limited, and data can only be obtained through a query interface. A query interface consists of a number of input attributes for users to set up their queries. An online database returns data that matches the query by generating web pages dynamically including one or more output attributes.

This paper focuses on the problem of outlier detection over the deep web. To the best of our knowledge, this problem has not yet been addressed in existing work. An outlier is an observation that deviates so much from other observations as to arouse suspicion that it was generated by a different mechanism. There is a great practical significance in detecting outliers over the deep web. For example, outliers may be commodities that have abnormal price because of mistakes during data entry. The third-party collaborators of a website want to detect outliers in time and notify the responsible person for the website to modify the data for the purpose of decreasing losses, while the website users have a great interest in finding these commodities.

Outlier detection has always been a hot research topic in the field of data mining. On this problem, a lot of exciting results have been published in recent years. As the survey [4] described, outlier detection techniques can be broadly divided into distance-based approaches [5, 6], density-based approaches [7, 8], clustering-based approaches [9], and information theoretic approaches [10]. However, these outlier detection methods need to know the distribution of underlying data, which is impractical in the context of deep web. According to the best of our knowledge, this is the first work on detecting outliers in deep web.

A naive solution for outlier detection in deep web is to download all the records from a back-end database and then mine its outliers using a traditional outlier detection approach discussed in the survey [4]. As stated earlier, records from the back-end database can only be retrieved by submitting queries to its corresponding query interface. Since queries consume server resources a lot of time and there exist top-*k* records constraints and query times constraint for every IP address, that is, restrictions over the query interface, this naive method is costly impractical.

Thus, a practical solution is to randomly sample the back-end database of a deep web to detect outliers. The back-end database is a kind of hidden database. Sampling for outlier detection in a hidden database has been studied in [11]. They proposed a random walk scheme over the query space provided by the interface to randomly sample such databases. Their method uses random sampling for addressing the problem. In their method, the computation between each data record is very expensive. Unlike their work, samples can only be obtained by querying a deep web in our scenario. The cost of submitted queries is much more expensive, comparing with computation or memory cost. Therefore, the sampling cost, which refers to the number of distinct queries to be issued to obtain a sample from the deep web, is the dominant factor of detecting outliers in the deep web. Furthermore, random sampling is often used as a baseline method in the statistical areas. As outliers are rare, random sampling will not achieve a good recall intrinsically. This indicates that the random sampling method needs a great amount of sample cost in order to detect reasonable outliers, which is not suitable in the context of deep web.

Our idea in this paper on outlier detection on deep web is primarily related to distance-based outlier detection. We formally define that an instance *i* is considered to be an outlier if the percentage of the instances in a database that lie greater than a given distance from the instance *i* is greater than a true percentage threshold. However, the true percentage value is unavailable. We have to estimate it from the sample we have obtained. As we know, instead of determining whether an instance is an outlier or not (i.e., a binary determination), it is much more suitable to assign each instance a probability of being an outlier. How to estimate the probability of an instance being an outlier is a difficult issue. In this paper, we estimate the probability of an instance to be an outlier based on its estimated percentage.

In summary, this paper first presents a completely novel problem: outlier detection in deep web. Then, it proposes and empirically evaluates a stratification-based outlier detection method over deep web. The detailed contributions of our solution can be concluded as follows. First, we present a completely novel problem: outlier detection in deep web. We have developed a stratification scheme for a deep web data source. In our method, the stratification is done through a hierarchical tree that models the relationship between the input and output attributes based on a pilot sample. Second, we have proposed a stratification-based outlier detection method over deep web. Instead of random sampling across the strata, we have developed a neighborhood sampling scheme for collecting more outliers. Query spaces with high probability of containing outliers are explored. Finally, we have developed an uncertainty sampling algorithm to verify the uncertain instances in order to improve the outlier detection precision.

The rest of this paper is organized as follows. In Section 2, we simplified the concept of deep web and outliers.  Section 3 elaborates the method we proposed to solve the problem of detecting outliers in the deep web. The comparison methods and experimental results are presented in Section 4.  Section 5 briefly reviews some related work. In Section 6, we give our concluding remarks.

## 2. Preliminary Concepts

Before we discuss our solution for outlier detection over deep web, we mainly introduce the basic process of sampling and outlier detection in the deep web environment in this section.

### 2.1. Process of Sampling in the Deep Web

Let us consider an example where Table 1 shows a part of a real deep web back-end database which contains 10 instances. Each column of Table 1 represents an attribute. The attribute can be divided into three types according to their domain, which are categorical, continuous, and text. The websites provide user with a query interface which contains a set of attributes what we call it the input attributes. The “Brand,” “Type,” and “Screen” attributes represent the three input attributes in our example. A user query can be looked as an assignment of a subset of input attributes of the query interface and the corresponding query results are returned in the form of HTML. For example, if a user queries the database with* Screen* = 13.3, then the 4th and 10th instances are returned as query results including the two output attributes* Price* and* StandBy*. In most cases, we are prone to mine the output attributes of interests, such that we are more likely to find abnormality prices in this example. The process of sampling the deep web is a repeated process of querying the back-end databases. It should be noted that there are a number of constraints in the query interface, such as the top-*k* constraints and IP restrictions. Therefore, a common objective for mining the deep web is to minimize the number of queries issued through the query interface.

### 2.2. DB-Outlier

Among the existing methods of detecting outliers, a distance-based outlier (DB-Outlier) detection is one of the most commonly used and simplest approaches. An object *p* in a dataset D is a DB(pct, d_min_) outlier if at least a percentage pct of the objects in D lie in the locations that are greater than distance d_min_ from *p*; that is, the cardinality of the set {*q* ∈ D∣*d*(*p*, *q*) ≤ d_min⁡_} is less than or equal to (100 − pct)% of the size of D. For example, when pct = 0.8 and d_min⁡_ = 600, the 9th instance would be an outlier if we only consider the price attribute as the interest output attribute.

According to our knowledge, outlier detection in the deep web is a completely novel problem. It engages outlier detection with the deep web. There is no existing solution available. In the following section, we will introduce our solution gradually.

## 3. Stratification-Based Outlier Detection

After having introduced the basic process of sampling and outlier detection in deep web environment, it is the time for us to elaborate the problem and our proposed method in this section. This paper primarily considers the case of categorical input attributes. Continuous input attributes can be considered as discrete categorical ones.

Typically, given a query composed of values of one or more of the input attributes, a deep web data source will return the number of data records satisfying the input query. Using this information, the distribution on the input attributes can be obtained. Since the distribution of the output attributes is unknown, discovering the outliers on the output attributes is a great challenge. However, if the relationship between the input attributes and the output attributes is known, we can identify the outliers of the output attributes with using the distribution of the input attributes. In our method, the relationship between the input attributes and the output attributes is built by stratification, which is a process of dividing an entire population into subpopulations based on a pilot sample.

There are two other important steps in our approach after stratification, which are neighborhood sampling and uncertainty sampling. The goal of these two sampling steps is to collect more outliers and keep a suitable precision under a limited cost. For each record we obtained, we assign it a probability of being an outlier. We classify each record into three classes (i.e., outlier, normal, and uncertain) based on its probability.

In general, our approach proceeds as follows. We obtain a pilot sample by randomly sampling the deep web first. Stratification of the population is conducted based on the pilot sample. Then, neighborhood sampling across the subpopulations is performed to collect more outliers. Next, in order to identify the uncertain ones, we perform uncertainty sampling so as to avoid the misjudgment. Each step of our approach will be explained in detail as follows.

### 3.1. Stratification

When the known population consists of several significantly differential parts, the population is always divided into subpopulations called strata for samples to adequately reflect the distribution of the population. In our algorithm, stratification is performed so that data records contained in the same stratum should be as similar as possible. Thus, we can isolate outliers in a few strata. The whole data in a deep web data source can be considered as the entire query space, whereas the subpopulations correspond to the query subspaces. After the stratification process, the distribution of output attributes is predicted effectively by the values of input attributes in each stratum. Query submission from the corresponding subspace can thus help us to obtain subpopulation data records. The primary purpose of stratification is to identify and group similar data records included in each stratum. Thus, how to perform stratification is an important issue.

We adopted the strategy of building a hierarchical tree to stratify the deep web data source. Formally, IS = {I_1_, I_2_,…, I_*s*_} represents the set of input attributes, {*a*_*j*,1_, *a*_*j*,2_,…, *a*_*j*,*m*_} represents the domain of I_*j*_, and OS = {O_1_, O_2_,…, O_*q*_} represents the set of output attributes of interests. For a leaf node, LN, let *Q* represent its corresponding query composed of SI, which is a subset of the input attributes. Under the query space of node LN, we define the radius for the corresponding subpopulation, *R*, which can be computed as follows:

(1) $$\begin{array}{c} R=\sqrt{\frac{\sum_{i}{\left({DR}_{i}\left(OS\right)-C\right)}^{2}}{N}}, \end{array}$$

where DR_*i*_(OS) = {DR_*i*_(O_1_),…, DR_*i*_(O_*q*_)} denotes a data record DR_*i*_ of the node LN's subpopulation and DR_*i*_(O_*j*_), *j* = 1,2,…, *q*, denotes the value for output attributes in DR_*i*_. *C* = ∑_*i*_(DR_*i*_(OS))/*N* is the center of the subpopulation corresponding to the node LN, and *N* is the size of the subpopulation corresponding to the node LN. For a deep web data source where the data is not directly accessible, the radius *R* is estimated based on a sample. Here, we introduce another concept that potential splitting input attributes PS are defined as the subset of input attributes that are not contained in the query *Q*; that is, PS = IS − SI. When stratifying a leaf node, we can choose an input attribute from PS.

For a potential splitting input attribute P_*i*_ ∈ PS associated with the domain DM_*i*_ = {*a*_*i*,1_,…, *a*_*i*,*t*_}, the decrease of radius is computed as

(2) $$\begin{array}{c} \Delta R_{i}=R-\overset{t}{\underset{k=1}{\sum }}p\left(\;P_{i}=\left(\;a_{i,k}\;|\;Q\;\right)\;\right)R_{i,k}, \end{array}$$

where *p*(P_*i*_ = *a*_*i*,*k*_∣*Q*) is the conditional probability of P_*i*_ that takes the *k*th value from its domain under the query space *Q*. The conditional probability can be computed as

(3) $$\begin{array}{c} p\left(\;P_{i}=a_{i,k}\;|\;Q\;\right)=\frac{\text{sel}\left(Q,a_{i,m}\right)}{\text{sel}\left(Q\right)}, \end{array}$$

where the function sel(*Q*) returns the number of data records under the space of *Q* in the back-end database, which is supported by most websites. We can even use the method proposed in the literature [12] to realize this function if it is not supported. The potential splitting input attribute P_*i*_ with the largest decrease of radius is chosen to split the space of the node LN. Using P_*i*_ to stratify the query space of LN, the node LN splits off *t* child nodes, where each node denotes a subpopulation. *R*_*i*,*k*_ denotes the radius for the *k*th child node generated by splitting input attribute P_*i*_ and its computation is similar to that of *R*. We then iterate the stratification on each child node. The query of the next iteration can generally be represented by *Q* = *Q* ∪ P_*i*_ = *a*_*i*,*k*_, *k* ∈ [1, *t*]. The whole process eventually forms a hierarchical tree where each leaf node represents a stratum.

In most cases, the input query space would be overstratified so that each stratum contains only one sample. Thus, we utilize a statistical hypothesis test to check whether the decrease of radius is significant. The idea behind the hypothesis test is that if there is not a significant relationship between the splitting input attribute P_*i*_ and the output attributes OS, the distribution of output attributes OS in the node LN would be similar to that in each of the children nodes. This means that there would be a little reduction in the radius after splitting the leaf node LN. Specifically, we use the *Z* hypothesis testing [13]:

 
*H*_0_: there is no significant relationship between P_*i*_ and the output attributes OS.
 
*H*_1_: there is a significant relationship between P_*i*_ and the output attributes OS.

The statistics *z* for the hypothesis testing is computed as

(4) $$\begin{array}{c} z=\frac{\left|\bar{R}-R\right|}{s/\sqrt{t}}, \end{array}$$

where *R* denotes the radius of the leaf node LN, $\bar{R}=\sum_{k=1}^{t}R_{i,k}/t$ denotes the average radius of child nodes of LN by splitting with the input attribute P_*i*_, *t* denotes the size of the domain of P_*i*_, and *s* ^2^ denotes the sample variance of the radius of child nodes. With a significant level *a*, the decision rule is as follows: if *z* > *z*_(1−*a*)/2_, we reject the hypothesis *H*_0_ and declare that there is a significant relationship between P_*i*_ and the output attributes OS. Otherwise, we accept the hypothesis *H*_0_ and declare that there is no significant relation between P_*i*_ and the output attributes OS. We can obtain the value of *z*_(1−*a*)/2_ from the standard statistics data.

In Algorithm 1, we summarize the overall process of splitting a node N, which is associated with the query *Q* and a list of potential splitting attributes PS. The input to the algorithm also includes a significant level *a* and a leaf node set LS. LS in our algorithm is used to save the leaf node in the tree. At the beginning, the entire query space is represented by the *ROOT* node. The corresponding query of the *ROOT* node is null and the potential splitting attributes list is the complete set of input attributes of this data source. The initial set of leaf nodes is empty.

For each potential splitting attribute, the radius decrement is computed according to (2) in Lines (1)–(10). The potential splitting attribute, which creates the maximum decrement in the radius, is selected, and its domain size is *t* (Line (10)). Following this, the radius of its each child node will be estimated according to (1) based on the pilot sample in Lines (11)–(14). Then, the statistical hypothesis test is conducted according to (4). If the null hypothesis is accepted, which means the radius decrement is not significant, the node N is set to a leaf node and will be included in the leaf node set. Otherwise, the space of node N is split by the *SplitAttribute*, and *t* children are generated for the node. The associated query of each child node N_*k*_ will be updated (Line (20)) and the set of potential splitting attributes is PS − *SplitAttribute* now. The process of splitting is then applied to the children nodes of node N in Line (21). The algorithm stops when there is no available node that can be further stratified.

### 3.2. Neighborhood Sampling

After stratification, similar data objects tend to be from the same query subspace or neighbor query subspaces. It indicates that we can obtain more outliers from the query subspace or its neighbor query subspaces in which we have identified outliers. Thus, the urgent key consideration is how to identify a data object's abnormality in the deep web. For a data object *d*, let *f* denote the fraction of data objects at distance greater than a given distance D. Combined with the definition of DB-Outlier we have described above, we can justify the data object *d* as an outlier if its fraction *f* is greater than a given threshold pct. As it is impractical to download all the data in the deep web, we have to estimate the fraction *f* based on the stratification.

Suppose that we have obtained *H* strata after stratification, given a sample S = {S_1_,…, S_*H*_} obtained from each stratum in the pilot sample; let *n*_*i*_ denote the number of samples drawn from the *i*th stratum; namely, *n*_*i*_ = |S_*i*_|. To facilitate our description, we define an indicator variable as

(5) $$\begin{array}{c} Z_{i,j}=\left\{\begin{array}{cc} 1, & \text{Dis}\left(d_{i,j},d\right)>D\\ 0, & \text{otherwise,} \end{array}\right. \end{array}$$

where *d*_*i*,*j*_ denotes the *j*th data record in the *i*th stratum and Dis⁡(*d*_*i*,*j*_, *d*) denotes a function for computing the Euclidean distance of a pair of data objects. Now, we introduce our estimation of the fraction *f* as

(6) $$\begin{array}{c} \tilde{f}=\overset{H}{\underset{i=1}{\sum }}p_{i}\frac{\sum_{j=1}^{n_{i}}Z_{i,j}}{n_{i}}, \end{array}$$

where *p*_*i*_ denote the proportion of the data amount of the *i*th stratum. It turns out that $\tilde{f}$ is an unbiased estimation; that is, $E(\tilde{f})=f$.

The variance for $\tilde{f}$ is

(7) Varf~=∑i=1Hpi2σi2ni,

(8) Varf~∈0,∑i=1Hpi214ni,

where *σ*_*i*_^2^ denotes the variance of indicator variable in the *i*th stratum.

According to (6), *p*_*i*_ and *n*_*i*_ are fixed with respect to the *i*th stratum. Therefore, the variance of $\tilde{f}$ can be written as

(9) Varf~∑i=1Hpi2ni2Var∑j=1nZi,j=∑i=1Hpi2niVarZi,j=∑i=1Hpi2niσi2.

Using the definition of *Z*_*i*,*j*_ in (5), it is shown that *Z*_*i*,*j*_ is a typical 0-1 distributed random variable. *Z*_*i*,*j*_ ~ *b*(1, *p*), where *p* is the probability of *Z*_*i*,*j*_ = 1. Thus,

(10) $$\begin{array}{c} \sigma_{i}^{2}=\text{Var}\left(\;Z_{i,j}\;\right)=p\left(\;1-p\;\right)\leq \frac{1}{4} \end{array}$$

only when *p* = 0.5 and *σ*_*i*_^2^ = 1/4. On combining the above, we immediately obtain (8).

Since query submitting is expensive, we need to collect more outliers with a low query cost. In other words, we want to retrieve outliers as much as possible within a given query cost. If the probability of each stratum containing outliers is given, we could achieve this goal easily by assigning more query cost to the stratum with a high probability.

For each data record DR in the sample S, we could identify the abnormality of DR by computing its fraction $\tilde{f}$. Thus, the probability of the *i*th stratum containing the outliers can be estimated as pro_*i*_ = *m*_*i*_/*n*_*i*_, where *m*_*i*_ is the number of outliers identified in the *i*th stratum. For the purpose of finding more outliers and keeping the underlying distribution, our method tends to allocate more samples to the stratum where its probability is higher or its population is large. With a given sample cost *t* across the strata, the sample allocation for the *i*th stratum *t*_*i*_ is computed as

(11) $$\begin{array}{c} t_{i}=\frac{p_{i}{\text{pro}}_{i}}{\sum_{j=1}^{H}p_{j}{\text{pro}}_{j}}t. \end{array}$$

This shows that the sample size of the *i*th stratum is proportional to its probability and population. It should be noted that there is a greedy fashion so as to maximize the size of the retrieved outliers. The greedy fashion works in the following way: sort strata in descending order according to its pro_*i*_ and then assign *N*_*i*_ to each stratum one by one until *t* samples have been collected, where *N*_*i*_ is the size of the population of the *i*th stratum. However, this greedy fashion will break the consistency between the sample distribution and the underlying distribution while our sampling allocation will not.

### 3.3. Uncertainty Sampling

In this subsection we introduce our uncertainty sampling method. The uncertainty here is with respect to the possibility for a data record belonging to the outliers. According to the degree of uncertainty, data records can be divided into three classes: (a) the outlier class; (b) the normal class; and (c) the uncertain class. For the data records belonging to (a) or (b) class, we can surely identify their abnormality. But for the data records belonging to (c) class, there is a great possibility of misjudgment occurring. The misjudgment, acknowledging true outlier as the normal class or true normality as the outlier class, will lead to a low precision. The fundamental reason for the misjudgment occurring is that there is a diversity between the distribution of samples and the underlying population, which leads to the incorrect estimation of the fraction. To solve this problem, we developed an uncertainty sampling with the goal of reducing the variance of estimated fraction $\tilde{f}$ in order to minimize the distance between $\tilde{f}$ and *f*.

Now, we formulate our description above. Let *p*(outlier) denote the probability of a data record to be an outlier. Thus, for a data record, we identify it as an outlier if *p*(outlier) > *θ*_1_ or a normal one if *p*(outlier) < *θ*_2_; otherwise we identify it as an uncertain one, where *θ*_1_, *θ*_1_ are the predefined parameters and 0 ≤ *θ*_2_ < *θ*_1_ ≤ 1. The computation of *p*(outlier) can be seen as the calculation of the probability of the fraction *f* transcending the threshold pct. According to the Lindburg-Levy theorem [14], the fraction *f* obeys the Gaussian distribution when the size of underlying population is large enough. As each data record has its estimated fraction $\tilde{f}$ and the variance $\text{Var}(\tilde{f})$, the probability distribution of the fraction has $f\sim N(\tilde{f},\sqrt{\text{Var}(\tilde{f})})$. As a result, the probability *p*(outlier) can be computed as

(12) $$\begin{array}{c} p\left(\;\text{outlier}\;\right)=p\left(\;f>\text{pct}\;\right)=1-\Phi \left(\;\frac{\text{pct}-\tilde{f}}{\sqrt{\text{Var}\left(\tilde{f}\right)}}\;\right), \end{array}$$

where Φ(·) is the standard Gaussian distribution function.

Thus, a set of uncertain data objects will be picked out. To improve the precision, our task is to obtain a sample for identifying uncertain data records. For uncertain data records US = {DR_1_,…, DR_*m*_} with estimated $\tilde{F}(US)=\{\tilde{f_{1}},\ldots ,\tilde{f_{m}}\}$, the summation variance of US is $\text{SumVar}(US)=\sum_{j=1}^{m}\left(\text{Var}(\tilde{f_{j}})\right)$. In this sampling phase, we need to decide the size of the sample drawn from the *j*th stratum so that the summation variance is minimized. As a result, the distance between estimated $\tilde{f}$ and *f* will be minimized. The total number of data records drawn from all the strata is fixed, which is denoted as *l* = ∑_*i*=1_^*H*^ *l*_*i*_. We formulate our goal as

(13) Minimize SumVarl1,…,lH=∑j=1mVarfj~subject  to ∑i=1Hli=l.

The solution for the objective to be minimized above is

(14) $$\begin{array}{c} l_{i}=\frac{p_{i}\sqrt{\sum_{j=1}^{m}\sigma_{i,j}^{2}}}{\sum_{r=1}^{H}\left(p_{r}\right)\sqrt{\sum_{j=1}^{m}\sigma_{r,j}^{2}}}l, \end{array}$$

where *σ*_*i*,*j*_^2^ denotes the variance of the indicator variable of the *i*th stratum with respect to the *j*th uncertain data record.

Using the definition of the summation variance, we have

(15) SumVarl1,…,lH∑j=1mVarfj~=∑j=1m∑i=1Hpi2σi,j2li=∑i=1Hpi2∑j=1mσi,j2li.

Under the limitation ∑_*i*=1_^*H*^ *l*_*i*_ = *l*, the problem of minimizing SumVar is a typical convex optimization problem in the field of statistics. Using the well-known optimization method* Lagrange* multipliers, we can directly obtain the solution described in (14).

Our uncertainty sampling method can be viewed as a generalization of the* Neyman* sample allocation method [15] for stratified sampling. The size of sample drawn from the *i*th stratum is proportional to the size of the subpopulation and the summation variance of the indicator variable. The distance between the estimated $\tilde{f}$ and *f* is minimized after uncertainty sampling. We now introduce our method of mining outliers from the uncertain data records.

A distance between each pair of sampled data records is computed and then we compute the probability *p*(outlier) for each sampled data record. A data record is determined to be an outlier if *p*(outlier) > *η*; otherwise it is a normal data record, where *η* is a predefined parameter.

A sufficient condition for identifying a data record is as follows.

For the outliers,

(16) Zη>0,f~>pct+Zη∑i=1Hpi214ni,Zη≤0,f~>pct.

For the normal data records,

(17) Zη>0,f~>1−pct,Zη≤0,nf~>1−pct−Zη∑i=1Hpi214ni,

where $\tilde{nf}$ denotes the estimation of fraction of neighbors in D neighborhood and *Z*_*η*_ denotes the quantile of the standard Gaussian distribution.

For a sampled data record DR, DR is identified as an outlier if *p*(outlier) > *η*; otherwise DR is identified as a normal data record. To facilitate our presentation, we use *nf* to denote the fraction of neighbors in distance D neighborhood; $\tilde{nf}$ denotes the estimated *nf* where $\tilde{nf}=1-\tilde{f}$ certainly. Using (12), we have the following.

If DR is an outlier,

(18) $$\begin{array}{c} \tilde{f}>\text{pct}+Z_{\eta }\sqrt{\text{Var}\left(\tilde{f}\right)}. \end{array}$$

Otherwise,

(19) $$\begin{array}{c} \tilde{nf}>1-\text{pct}-Z_{\eta }\sqrt{\text{Var}\left(\tilde{f}\right)}, \end{array}$$

where *Z*_*η*_ denotes the quantile of the standard Gaussian distribution. *Z*_*η*_ ≤ 0 when 0 < *η* ≤ 0.5, and *Z*_*η*_ > 0 when 0.5 < *η* ≤ 1. With the knowledge of (8), we then conclude the sufficient condition described in formulas (16) and (17).

### 3.4. Summary: Overall Algorithm

Now, we summarize our overall method for detecting outliers from a deep web data source. The overall process is shown in Algorithm 2. The inputs for the algorithm are the set of input attributes IS, the set of interested output attributes OS, the size of sample *n*_nb_ that is to be drawn in the neighborhood sampling, and the size of sample *n*_u_ that is to be drawn in the uncertainty sampling.

At the beginning, a pilot sample is drawn from the entire population of the deep web data source by random sampling [11]. The entire query space of the deep web is stratified by calling Algorithm 1 from the root node (Lines (1)–(4)). A stratum is represented by a leaf node LN as we described before. After stratification, the samples in the pilot sample can be divided into *H* strata, which is S_1_,…, S_*H*_. Thus, we estimate the probability of containing outliers for each stratum based on its samples (Line (5)). Next, the neighborhood sampling is performed in the following steps: for each leaf node LN_*i*_, the number of data records *n*_*i*_, to be drawn from the space of LN_*i*_, is computed using (11) in Lines (6)–(10). After obtaining the neighborhood samples, we can identify the outliers and the uncertain data records US by computing their estimated fraction (Lines (11)–(19)). Next, we conduct the uncertainty sampling for US. For each leaf node LN_*i*_, the number of data records *n*_*i*_, to be drawn from its query space, is computed by (14) in Lines (20)–(24). After obtaining these data records, we finally mine the outliers from the data records which are not identified as outliers before and combine the outliers as our final result.

## 4. Experimental Evaluations

In this section, we will evaluate the benefits from using the stratification strategy over the query space, neighborhood sampling, and uncertainty sampling, respectively, and compare our proposed method with the baseline on the deep web using three different datasets. (1) A synthetic dataset is generated by MATLAB. (2) HTTP and SMTP are the subsets of KDD CUP 1999 that could be downloaded from the UCI repository; HTTP and SMTP are benchmark datasets for outlier detection. (3) A live experiment is conducted on https://autos.yahoo.com/. In order to evaluate the benefit of each component of our solution, we create several variants of our solution in the following subsection.

### 4.1. Setup

Our evaluation has been performed over a combination of real and synthetic datasets.

Our synthetic dataset is generated by MATLAB. It contains 4100 data records, including 4000 normal records and 100 outlier records. There are seven attributes (i.e., 5 categorical input attributes and 2 continuous output attributes). Four clusters exist on the two output attributes, which are generated by a Gaussian distribution. The output attributes are created to be dependent on the input attributes.

Two real datasets, referred to as HTTP and SMTP, are the subsets of KDD CUP 1999 that could be downloaded from the UCI repository. The HTTP dataset contains 623091 data records while the SMTP dataset contains 96554 data records. Preprocessing has been performed on these two datasets. We randomly sample 8000 data records from these two datasets, respectively, as our final experimental datasets. The original dataset contains 41 attributes, whereas we only reserve two basic attributes (i.e., “*src_bytes*” and “*dst_bytes*”) as our output attributes and five attributes (i.e., “*duration*”, “*flag*”, “*land*”, “*wrong_fragment*”, and “*urgent*”) as our input attributes. The repeated data in each dataset is reserved only once. Instead of using the existing abnormal labels of data records, we prefer to label the data records utilizing the concept of DB-Outlier.

We conduct live experiments over a subset of a real-word hidden database (https://autos.yahoo.com/) of the data on new cars located within New York, NY, and Washington, DC. The deep web database consists of 3 attributes (Make, Model, and Zip) as input attributes and 7 attributes (i.e., brand, distance, mileage, year, price, etc.) as output attributes.

In our experiments, we set the significant level, where the corresponding statistics is 1.96, at the step of overstratification within the stratification. We adopt the three common criteria to evaluate all methods, which is precision, recall, and *F*-measure. Here, we briefly introduce the three criteria. Precision is the percentage of the true outliers within the whole outliers identified by our method. Recall is the percentage of the true outliers within the whole underlying outliers. The criterion *F*-measure is the geometric mean of precision and recall, which is commonly used in the unbalanced classification. As the characteristics of outliers, the criterion *F*-measure is very suitable in our scenario.

Outlier records of synthetic datasets HTTP and SMTP are known in order to evaluate our methods on live deep web database https://autos.yahoo.com/, which are precision, recall, and *F*-measure. We download the data on new cars located within New York, NY, and Washington, DC. This yields 163,000 data records. The data consists of 7 attributes (i.e., brand, distance, mileage, year, price, etc.). Then we use outlier detection tools to detect outlier records of the dataset, as the experimental evaluation benchmarks.

For each dataset, we repeat the independent experiments 50 times, and the average results are reported as our final results. The *x*-axis of our experimental figures represents the total sample size *s*. The size of pilot sample is always *s*/2 and the ratio between neighborhood sampling and uncertainty sampling is always 1.

Our proposed method, referred to as SNU, will be compared with the following methods:

- SRS: we refer to the simple random sampling method as SRS. Compared with our proposed algorithm, this method works in a manner that uses random sampling in each sampling step. This method is a baseline method.
- SN: this method is similar to our proposed algorithm except that it uses the random sampling replacing the uncertainty sampling.
- SU: this method is similar to our proposed algorithm except that it uses the random sampling replacing the neighborhood sampling.

### 4.2. Evaluation of Stratification and Neighborhood Sampling

In this subsection, we focus on evaluating the benefits from stratification and neighborhood sampling. For this purpose, we compare our method SNU with two other methods, SRS and SU.

**Figure 1:**
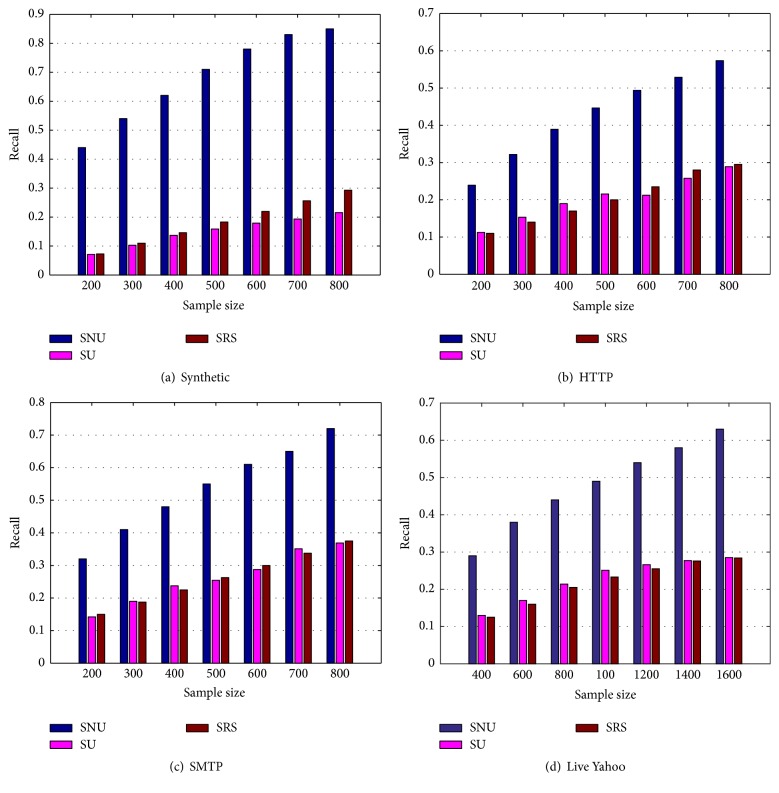
Evaluation of stratification and neighborhood sampling (recall).

Figures 1(a), 1(b), 1(c), and 1(d) show the comparisons among the three methods in terms of recalls on the four datasets (one synthetic dataset, two real datasets: HTTP and SMTP, and one live Yahoo dataset). From Figure 1(a), we can see that the recalls of both SU and SRS are significantly smaller than that of SNU. This demonstrates the effectiveness of neighborhood sampling, the component in our proposed method SNU. This also shows that the distribution of the input attributes is helpful for collecting more outliers, where the stratification on the population is necessary for neighborhood sampling. Furthermore, comparing with SRS, SNU improves its recall by up to 163.1%. This provides us with the confidence of applying our method to real-world deep web applications to detect outliers.

Figures 1(b) and 1(c) show the results on the two real datasets. There is a very similar trend in the results. Compared with the synthetic dataset, all methods have lower recall. A reasonable explanation is that the relationship between the input attributes and the output attributes is not as strong as for the synthetic dataset. As a result, the similarity of data records contained in the same stratum is not high. After careful observation, we can find that the recall in the SMTP dataset is higher than that in the HTTP dataset under the same sample size. This is likely because the outlier rate in the SMTP is correspondingly higher than in the HTTP. Moreover, compared with SRS, SNU has improved the recall by up to 109.7% average in these two real datasets.

The remaining Figure 1(d) shows the results on the live deep web database https://autos.yahoo.com/. There is also a very similar trend in the results. Compared with the three datasets, all methods have lower recall under the same sample size. Moreover, compared with SRS and SU, SNU has improved the recall by up to 111.6%, 119.7% average in the live dataset. Thus, our approach clearly results in more effective methods than using SU and SRS on the live deep web.

### 4.3. Evaluation of Uncertainty Sampling

We now evaluate the benefits of uncertainty sampling to further identify the uncertain data records. For this purpose, we compare our methods with two other methods, SRS and SN.

**Figure 2:**
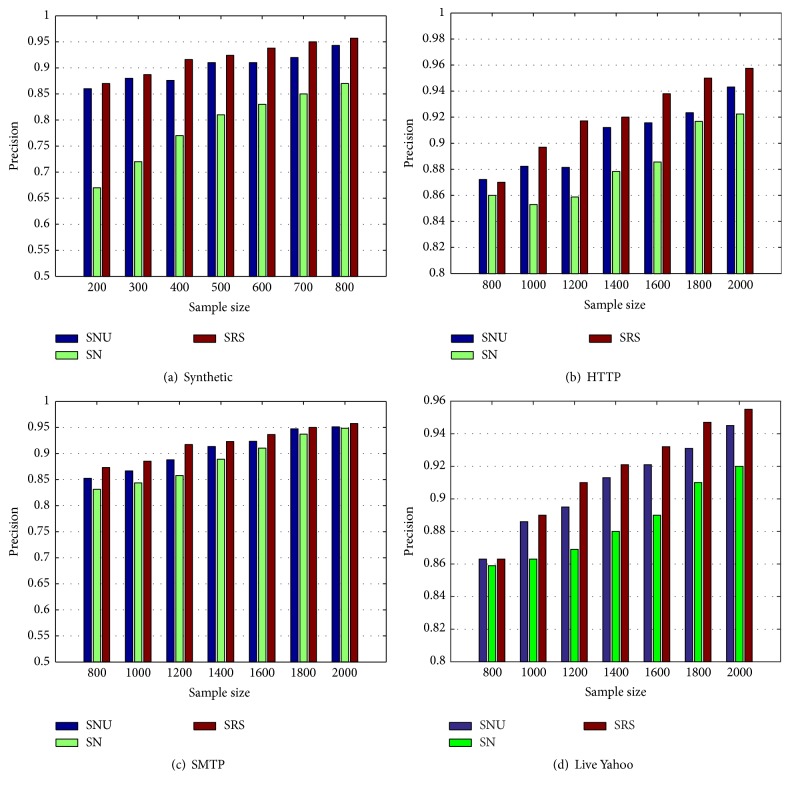
Evaluation of uncertainty sampling (precision).

Figure 2 shows the comparison of the precision for these three methods, applied on the four datasets.  Figure 2(a) shows the results from the synthetic dataset while Figures 2(b), 2(c), and 2(d) show the results from the HTTP, SMTP, and live Yahoo datasets, respectively. Figures 2(a), 2(b), 2(c), and 2(d) show that the precision of SN is smaller than of SNU. This demonstrates the effectiveness of uncertainty sampling because SN uses random sampling instead of uncertainty sampling. However, the precision of our method SNU is a little lower than that of SRS. This is mainly due to the fact that the output attributes distribution of our method has a bias after neighborhood sampling. It should be noted that this situation can be alleviated when the sample size is large enough. Furthermore, we can also see that both SNU and SRS have convincing precisions on all four datasets. This indicates that we do not need to spend much effort in improving the precision. However, we do need to pay more attention.

### 4.4. Evaluation of Detection Performance

**Figure 3:**
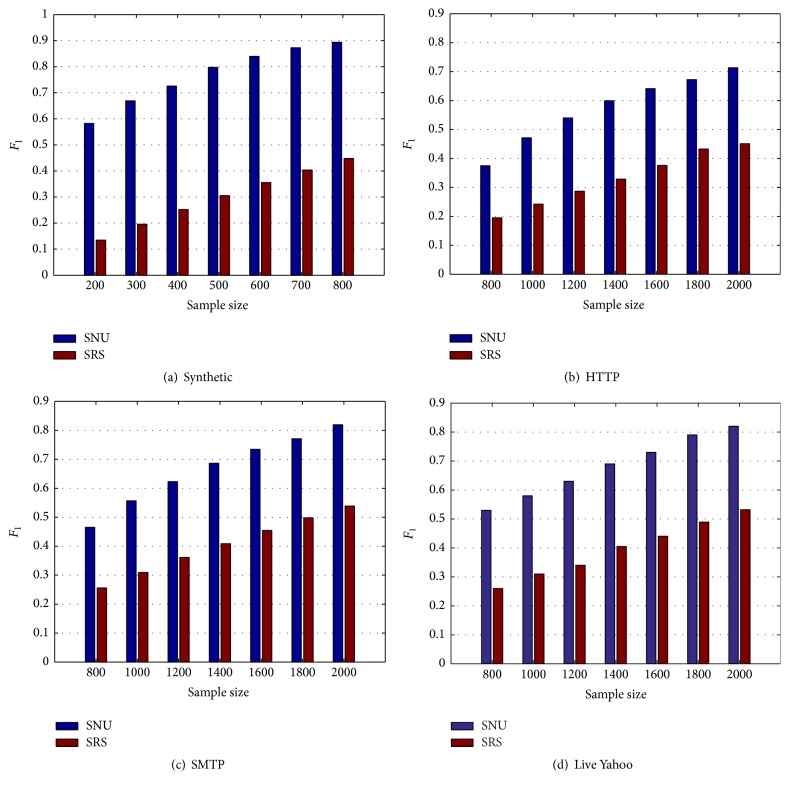
Evaluation of detection performance (*F*-measure).

After evaluating the benefits of each component of our method SNU, we will evaluate the performance of our method SNU in terms of detecting outliers over deep web. For this purpose, we compare our method with the baseline method SRS on the four datasets.  Figure 3 shows that our method outperforms SRS method in all datasets. The average improvement of *F*-measure in the synthetic, HTTP, SMTP, and live Yahoo datasets is 184.5%, 78%, 68.1%, and 75.4%, respectively. Overall, we observe that our method needs fewer sampled data records over SRS to achieve the same performance. Since the cost of obtaining each sample is the dominant factor in mining the deep web, this reflects that our method can achieve significant reductions in the query cost. Furthermore, combined with the corresponding recalls and precisions in Figures 1 and 2, we conclude that recall is the dominant factor in improving the performance of outlier detection methods over deep web. In addition, precision has little room for improvement.

## 5. Related Work

There exists a vast majority of research on the deep web. However, these researches focus on how to build an interactive query system or a vertical search system using data integration technologies [16–18]. Recently, with the development of sampling and crawling over the deep web [19–21], mining deep web has attracted more attention than before [22–24]. Moreover, outlier detection research has always been a hot topic in machine learning and data mining. However, existing work has not considered the combination of these two aspects yet. According to our knowledge, this paper is the first to conduct outlier detection over the deep web. In this section, we will present the related work and discuss the difference of our work next.

### 5.1. Sampling for Outlier Detection

Outlier detection using a sampling strategy has been described by various researchers [25–27]. Wu et al. utilized a sampling method to detect DB-Outliers. They assumed that the calculation cost of the distance between each pair of samples is expensive. Instead of calculating the distance between each pair of samples, their method randomly samples samples from the neighborhood points. Kollios et al. attempted to identify a density estimator based on random sampling and then used the density estimator to detect DB-Outliers with biased sampling [26]. Abe et al. presented a classification-based outlier detection method in their study [27]. They attempted to transform an outlier detection problem into a simplified classification problem based on a sample selection mechanism of active learning. Unlike their work background, the problem we are considering in this paper is in the context of deep web. Even if a particular sample we need has to be collected, we cannot obtain it directly because of the characteristics of deep web.

### 5.2. Deep Web Sampling

Dasgupta et al. [11] proposed a random sampler called HDSampler. They attempted to obtain a random sample set from a deep web by utilizing a random walk-over-input-attribute space strategy. The research of Liu and Agrawal [28] is relatively similar to our work. They addressed the problem of clustering over a deep web data source by stratified sampling [29–31]. Their method performed stratification over the deep web first, then selected the representative sample from each stratum, and finally conducted hierarchical clustering. Inspired by their work, we proposed a method addressing the problem of outlier detection over a deep web data source in this paper.

## 6. Conclusions

This paper presents a novel problem in deep web: outlier detection over deep web. In this paper, we proposed a novel solution that can divide the outlier detection procedure into three components: stratification over deep web, neighborhood sampling, and uncertainty sampling. We developed the stratification scheme through a hierarchical tree to model the relationship between the input attributes and the output attributes. Instead of random sampling across the strata, we developed a neighborhood sampling scheme for collecting more outliers. Furthermore, we developed the uncertainty sampling algorithm to verify the uncertain instances in order to improve detection precision. We evaluated the performance of our solution empirically via the synthetic and real datasets. Our experimental results show that our approach indeed enhances significantly recall and *F*-measure, comparing with the simple random sampling method. In the future, we wish to address this problem by mapping a deep web data source into a graph and conduct data mining over the graph.

## Figures and Tables

**Algorithm 1:**
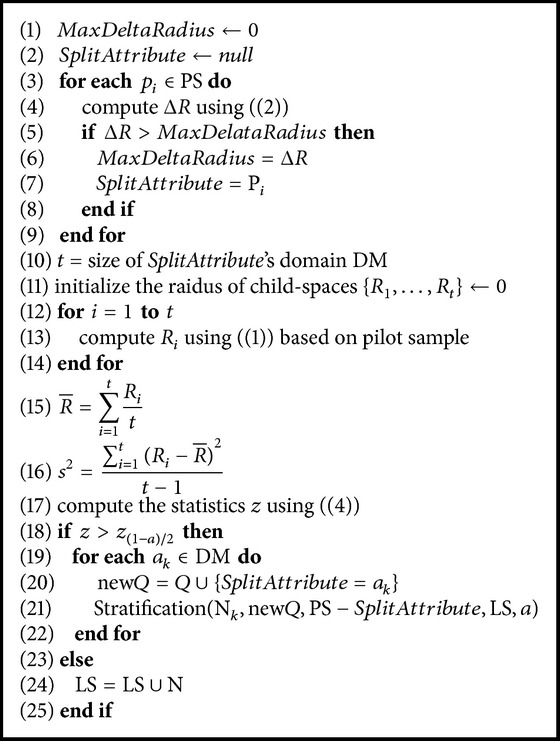
Stratification(N, *Q*, PS, LS, *a*).

**Algorithm 2:**
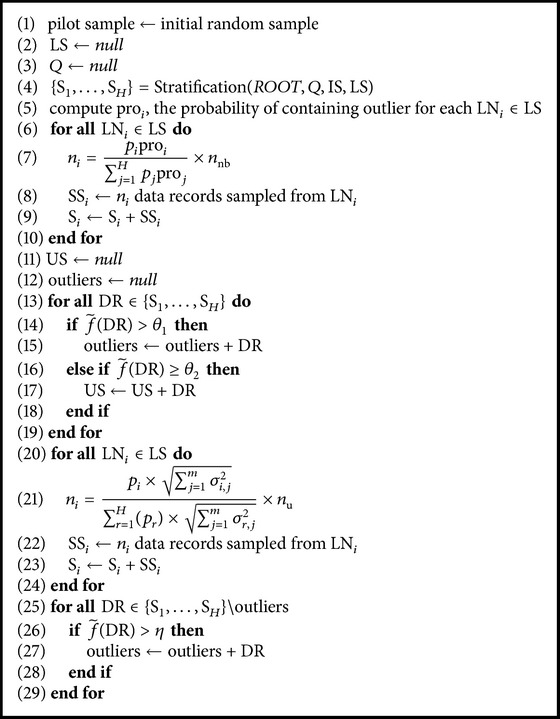
DeepDetection(IS, OS, *n*_nb_, *n*_u_).

**Table 1:** An example of a back-end database for electronic products.

ID	Brand	Type	Screen (inch)	Price ($)	StandBy (h)
1	Samsung	Phone	3.5	666.51	36
2	Samsung	Phone	3.5	356.06	30
3	Samsung	Phone	4.3	378.8	30
4	Samsung	Laptop	13.3	666.51	6
5	Samsung	Laptop	11.6	1107.53	8
6	Apple	Phone	4.0	801.21	36
7	Apple	Phone	4.0	696.81	36
8	Apple	Phone	3.5	498.18	24
9	Apple	Laptop	15.4	2831.51	10
10	Apple	Laptop	13.3	1180	10
